# Imaging characteristics of Intrasellar cavernous hemangioma: A case report

**DOI:** 10.1097/MD.0000000000023405

**Published:** 2020-11-20

**Authors:** Xinfa Pan, Jie Shen, Yuehui Ma, Haiyan Lou, Yuxiang Weng, Renya Zhan

**Affiliations:** a Department of Neurosurgery; b Department of Radiology, The First Affiliated Hospital, College of Medicine, Zhejiang University, Hangzhou 310003, Zhejiang, China.

**Keywords:** extra-axial hemangioma, cavernous sinus, sellar region, diagnosis, surgery

## Abstract

**Rationale:**

Intrasellar cavernous hemangiomas (ICHs) are rare vascular lesions that arise in the sellar region. ICHs are usually misdiagnosed and treated as pituitary adenomas. Therefore, a preoperative diagnosis is particularly important, especially when the goal is complete resection.

**Patient concerns:**

A 55-year-old woman presented with a 1-month history of intermittent dizziness. Magnetic resonance imaging (MRI) revealed a well-demarcated abnormal ellipsoid signal in the sellar region (size: 2.7 cm × 1.7 cm), with a mulberry-like enhancement after gadolinium injection. Computed tomography revealed an intrasellar mass without calcification that extended into the left cavernous sinus and was faintly contrast-enhanced. Angiography revealed a tumor with mildly delayed staining fed by the C5 segment of the right internal carotid artery.

**Diagnosis:**

An intrasellar cavernous hemangioma based on neuroradiological examinations.

**Interventions:**

The patient underwent surgery with an endoscopic endonasal transsphenoidal approach to debulk the lesion and obtain tissue for the pathological diagnosis.

**Outcomes:**

Blood spurting was observed after puncture, and the capsule was stained blue. Lesion removal was stopped, and the patient underwent gamma knife surgery 1 week later. She remained in good condition during the follow-up.

**Lessons:**

Sponge-like or mulberry-like lesions can be identified on MRI after gadolinium injection and can facilitate a preoperative diagnosis of ICH. Currently, surgical debulking with cranial nerve decompression during the acute stage and subsequent gamma knife radiosurgery are considered to be a safe and effective treatment.

## 1 Introduction

Cavernous hemangiomas are benign vascular lesions that arise in both the intra- and extra-axial regions of the brain. The incidence of this tumor type is 0.4% to 0.8% in the general population.^[1,2]^ Intrasellar (ICH) and parasellar cavernous hemangiomas are the most common extra-axial forms, although both types (especially ICH) are considered extremely rare.^[3,4]^ The first case of extra-axial cavernous hemangioma was reported in 1980 in a 72-year-old black woman with a metastatic breast carcinoma whose tumor was found incidentally during an autopsy.^[5]^ Successive cases of ICHs have been reported due to advances in imaging technology and particularly magnetic resonance (MR) applications.^[4,6–13]^ ICH has a characteristic appearance on MR imaging (MRI), including isointensity on T1-weighted images, hyperintensity on T2-weighted images and diffuse homogeneous enhancement on gadolinium-enhanced T1-weighted images in the sellar region. Still, these lesions are frequently misdiagnosed and treated as pituitary adenomas.^[14,15]^ To date, most studies of ICH involve case reports, which differ in terms of the imaging performance and typing of the disease (origin, expansion, and pattern of growth). In this report, we present a case of ICH with a comprehensive imaging examination, including MRI, computed tomography (CT), and digital subtraction angiography (DSA), and summarize the findings of previous case reports to yield a reference that may facilitate preoperative diagnoses.

## 2 Case report

**Figure 1:**
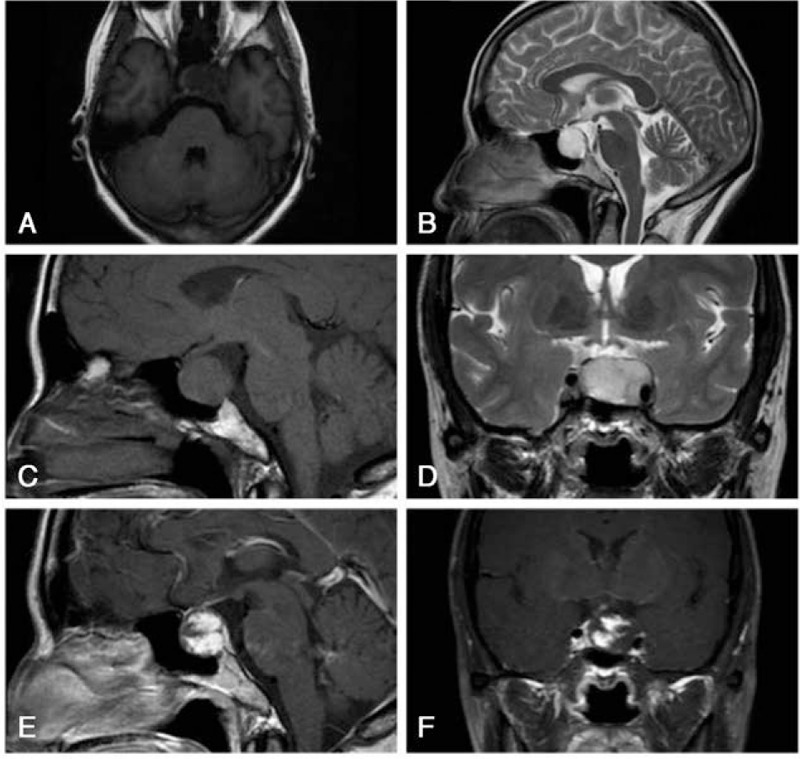
Magnetic resonance image showing a mass lesion in the sellar region with a size of 2.7 cm × 1.7 cm and a clear boundary. The lesion is located mostly in the intrasellar region but had pushed the optic chiasm slightly upward and invaded the left cavernous sinus. It appeared isointense on T1-weighted images (a and c) and hyperintense on T2-weighted images (b and d). Gadolinium contrast revealing heterogeneous enhancement with a mulberry-like appearance after gadolinium injection (e and f).

A 55-year-old woman presented with a 1-month history of intermittent dizziness, which could be relieved by rest and was not accompanied by nausea, vomiting, and sight rotation. She was otherwise healthy and had no history of chronic diseases such as hypertension and diabetes. She was initially diagnosed at a neurology clinic and was referred for a whole-brain MR scan, which revealed an intrasellar mass (Fig. 1a and b). The patient was referred to our neurosurgery department for further diagnosis and treatment.

Upon admission, she was asymptomatic other than intermittent dizziness. A physical examination revealed no positive signs, and an endocrinological evaluation revealed normal pituitary function. A dynamic enhanced MR scan of the pituitary revealed an abnormal ellipsoid signal in the sellar region with dimensions of 2.7 cm × 1.7 cm and a clear boundary. The lesion was located mostly in the intrasellar region but had pushed the optic chiasm slightly upward and invaded the left cavernous sinus. It appeared isointense relative to the gray matter on T1-weighted images (Fig. 1a and c) and hyperintense on T2-weighted images (Fig. 1b and d). An injection of gadolinium contrast revealed heterogeneous enhancement with a mulberry-like appearance (Fig. 1e and f), consistent with a well-vascularized lesion.

**Figure 2:**
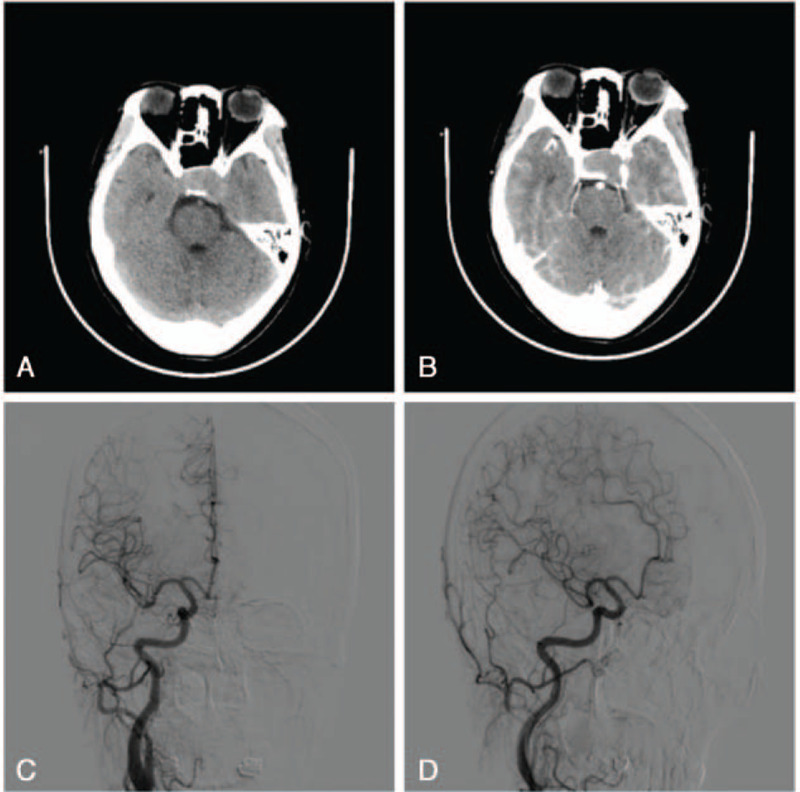
Computed tomography showing an intrasellar mass extending into the left cavernous sinus without calcification (a) and faint contrast enhancement (b). Right internal carotid angiography revealing a tumor with mildly delayed staining that was fed by the C5 segment of the internal carotid artery (c and d).

Next, the patient underwent CT angiography (CTA) and DSA to determine whether the lesion was a thrombotic aneurysm. A CT scan revealed an intrasellar mass without calcification that extended into the left cavernous sinus and exhibited faint contrast enhancement (Fig. 2a and b). Right internal carotid artery (ICA) angiography revealed a tumor with mildly delayed staining that was fed by the C5 segment of the ICA (Fig. 2c and d). No obvious aneurysms or draining vessels were visualized. A preoperative diagnosis of ICH was made based on these neuroradiological examinations.

**Figure 3:**
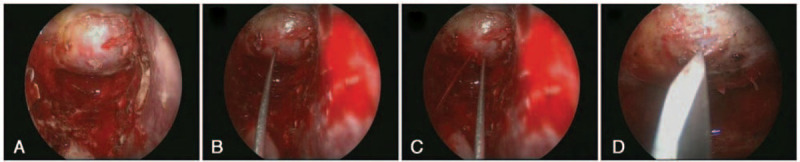
Intraoperative findings. The meningeal tension was high (a). The mass was punctured (b), but red blood was ejected when the puncture needle was removed (c). A blue-stained tumor envelope was visible after the meningeal window was opened (d).

The patient underwent surgery via the endoscopic endonasal transsphenoidal approach to debulk the lesion as much as possible and obtain tissue for a pathological diagnosis. Upon opening the sellar floor, tensioned meninges were encountered and the pulsation was visible (Fig. 3a). We first punctured the mass in the saddle (Fig. 3b). However, red blood was ejected when the puncture needle was pulled out (Fig. 3c). A blue-stained tumor envelope was visible after the meningeal window was opened (Fig. 3d). The lesion resection surgery was stopped due to the risk of excessive bleeding and sensitivity to radiation therapy. Though without pathological diagnosis, the patient received gamma knife therapy 1 week later. The patient remained in good condition and the MRI showed no obvious changes in lesion volume after 2 year follow-up.

## 3 Discussion

ICH is a rare type of extra-axial cavernous hemangiomas. These lesions share histological features with intra-axial lesions but have a different clinical manifestation and are usually misdiagnosed as pituitary adenomas. Moreover, resection is associated with high morbidity and mortality.^[16]^ We have provided a table in which the characteristic clinical manifestations, imaging features, and treatment methods are described clearly with the aim of better understanding the characteristics of pituitary adenomas, ICHs, cavernous sinus hemangiomas and cerebral cavernous hemangiomas (Table 1). The clinical symptoms of ICHs are attributable to the anatomical positions of the sellar region and the pituitary and cavernous sinuses and include headache, dizziness, cranial neuropathy, and endocrinopathy. These symptoms are relatively unique, although cranial neuropathy and epilepsy are also present in a subset of the cases of cavernous sinus hemangioma. However, both ICH and cavernous sinus hemangioma can be asymptomatic.^[2,17]^ Hemorrhage is the most common manifestation of a cerebral cavernous hemangiomas, followed by epilepsy and focal neurological deficits according to the location of the hemangioma. Some asymptomatic cerebral cavernous hemangiomas are usually discovered incidentally during a physical examination.^[2,18]^

**Table 1:** Characteristic of pituitary adenomas, intrasellar cavernous hemangiomas, cavernous sinus hemangiomas, and cerebral cavernous hemangiomas.

		Pituitary adenomas	Intrasellar cavernous hemangiomas	Cavernous sinus hemangiomas	Cerebral cavernous hemangiomas
Clinical manifestations		headache and vision defect	headache and dizziness	cranial neuropathy	hemorrhage
		endocrinopathy	endocrinopathy	headache and dizziness	incidental discovery
		cranial neuropathy	cranial neuropathy	epilepsy	epilepsy
		asymptomatic	asymptomatic	asymptomatic	focal neurological deficit
Imaging features	MRI	isointense on T1 and T2, homogeneous enhancement.	isointense on T1, markedly hyperintense on T2, homogeneous or heterogeneous enhancement.	isointense on T1, markedly hyperintense on T2, homogeneous or heterogeneous enhancement.	mixed signal intensity core with heterogeneous enhancement and a rim of hemosiderin
	CT	Isodensity or hyperdensity, mild or homogeneous enhancement.	Hyperdensity, mild or homogeneous enhancement.	hyperdensity, mild or homogeneous enhancement.	hyperdensity to isodensity, accompanying by mild hemorrhage or calcification
	DSA	avascular without feeding and draining vessel	avascular or vascular with feeding and draining vessel	avascular or vascular with feeding and draining vessel	abnormal drainage vein (occasionally)
Treatment methods		Medical treatement, surgery or observation.	surgery, radiosurgery or a combination of both.	surgery, radiosurgery or a combination of both.	observation, surgery, and radiosurgery depending on the clinical presentation and anatomic location.

MRI = magnetic resonance image CT= computer tomography, DSA = digital subtraction angiography.

The preoperative radiological diagnosis of an ICH is a crucial determinant of the treatment program. Therefore, the imaging features must be understood, as these can distinguish ICHs from other common lesions in the sellar region. First, significant differences have been observed between the imaging features of extra-axial and intra-axial cavernous hemangiomas, the later are surrounded by a ring of hypointensity due to hemosiderin deposits from recurring microhemorrhages. The specific imaging features of cerebral cavernous hemangiomas are described in Table 1. Second, many case reports of extra-axial cavernous hemangiomas have emphasized the specificity and sensitivity of a radiological diagnosis based on MRI, although such studies are not sufficiently comprehensive.^[4,11,12]^ Although most reports have described the characteristic MRI features (isointensity on T1-weighted images and hyperintensity on T2-weighted images), the gadolinium-enhanced T1-weighted images can be classified into 2 completely different manifestations.^[19,20]^ Specifically, Zhou reported 2 types of cavernous sinus hemangioma: sponge-like (bright homogeneous enhancement) and mulberry-like type (heterogeneous enhancement).^[21]^ The enhancement form in our case was the extremely rare mulberry-like type. Turan first reported this marked hyperintensity on T2-weighted images and the presence of signal void areas in an ICH patient.^[11]^ Third, CT and DSA are also important radiological examinations for the diagnosis of an extra-axial cavernous hemangiomas, although the manifestations are not specific and not widely reported. Only 2 cases have reported the DSA features of extra-axial cavernous hemangiomas in the literature.^[9,22]^ In one case, angiography demonstrated an avascular mass with no feeding or draining vessels. In the other case, an angiogram revealed a highly vascular mass that was fed from both anterior cerebral arteries and drained through the cavernous sinus. CT is used more commonly in early cases, and most of them exhibit hyperdense lesions with homogeneous or mild enhancement.^[13]^ The case reported here involves the first comprehensive imaging examination of ICH with MRI, CT, and DSA findings.

Intrasellar and parasellar extra-axial cavernous hemangiomas can be classified into 3 types according to the relationships among the tumor origin, expansion, and ICA on coronal MRI.^[17]^ According to this classification, many ICHs reported in the literature belong to the mixed type (Type III), while only a few cases belong to the true intrasellar type (Type I). Lombardi divided sellar and parasellar extra-axial cavernous hemangiomas into 3 types based on the patterns of growth, namely endophytic lateral growth, endophytic medial growth, and exophytic growth.^[3]^ However, tumor growth is a dynamic process, and it can be difficult to judge the growth pattern based on a certain MR performance, especially when the tumor is large and spans multiple anatomical regions. Therefore, we believe that a classification based on the tumor morphology and anatomical location may help to predict the clinical course of the tumor during development, as well as the response to treatment.

Cavernous hemangioma is a benign tumor, and total removal is the optimal treatment option. However, surgery requires balancing the benefits with the natural history of the lesion and functional impairment, even in a case of cerebral cavernous hemangioma in which 50% is located in the cerebral hemispheres. Technologies such as functional MRI and neuro-navigation have increasingly enabled the safe removal of cerebral cavernous hemangiomas, including brainstem cavernous hemangiomas.^[23]^ However, remarkable intraoperative bleeding and postoperative neurological dysfunction are common results of the surgical treatment of ICHs and cavernous sinus hemangiomas. Consequently, only a few cases in the literature describe total resection.^[24]^ Surgical debulking with acute cranial nerve decompression, followed by gamma knife radiosurgery, is considered a safe and effective treatment option for ICH.^[2,4,17]^ Radiosurgery can also be performed directly (i.e., without biopsy) in some cases that can be diagnosed based on imaging findings.^[17]^

## 4 Conclusion

ICHs and cavernous sinus hemangiomas are rare extra-axial cavernous hemangiomas that can be classified into 3 types according to the relationships between the tumor origin, expansion, and ICA features on coronal MRI. ICHs belong to type I, which is confined medially to the ICA, and these lesions do not expand beyond the so-called carotid line. Gadolinium injection reveals either a sponge-like or mulberry-like appearance on MRI. Therefore, MRI can facilitate a preoperative diagnosis of ICH. Surgical debulking with acute-stage cranial nerve decompression, followed by gamma knife radiosurgery, is considered a safe and effective treatment for ICH.

## Author contributions

**Investigation:** Yuxiang Weng.

**Supervision:** Yuehui Ma, Haiyan Lou.

**Writing – original draft:** Xinfa Pan, Jie Shen.

**Writing – review & editing:** Renya Zhan.
